# Transcriptional profile of human thymus reveals IGFBP5 is correlated with age-related thymic involution

**DOI:** 10.3389/fimmu.2024.1322214

**Published:** 2024-01-22

**Authors:** Xiaojing Yang, Xichan Chen, Wei Wang, Siming Qu, Binbin Lai, Ji Zhang, Jian Chen, Chao Han, Yi Tian, Yingbin Xiao, Weiwu Gao, Yuzhang Wu

**Affiliations:** ^1^College of Bioengineering, Chongqing University, Chongqing, China; ^2^Institute of Immunology People’s Liberation Army (PLA) & Department of Immunology, College of Basic Medicine, Army Medical University (Third Military Medical University), Chongqing, China; ^3^Department of Cardiovascular Surgery, the Second Affiliated Hospital, Army Medical University, Chongqing, China; ^4^Organ Transplantation Center, the First Affiliated Hospital of Kunming Medical University, Kunming, Yunnan, China; ^5^Institute of Medical Technology, Peking University Health Science Center, Beijing, China

**Keywords:** IGFBP5, thymus involution, single-cell RNA sequencing, thymic epithelial cell, thymocyte

## Abstract

Thymus is the main immune organ which is responsible for the production of self-tolerant and functional T cells, but it shrinks rapidly with age after birth. Although studies have researched thymus development and involution in mouse, the critical regulators that arise with age in human thymus remain unclear. We collected public human single-cell transcriptomic sequencing (scRNA-seq) datasets containing 350,678 cells from 36 samples, integrated them as a cell atlas of human thymus. Clinical samples were collected and experiments were performed for validation. We found early thymocyte-specific signaling and regulons which played roles in thymocyte migration, proliferation, apoptosis and differentiation. Nevertheless, signaling patterns including number, strength and path completely changed during aging, Transcription factors (FOXC1, MXI1, KLF9, NFIL3) and their target gene, IGFBP5, were resolved and up-regulated in aging thymus and involved in promoting epithelial-mesenchymal transition (EMT), responding to steroid and adipogenesis process of thymic epithelial cell (TECs). Furthermore, we validated that IGFBP5 protein increased at TECs and Hassall’s corpuscle in both human and mouse aging thymus and knockdown of IGFBP5 significantly increased the expression of proliferation-related genes in thymocytes. Collectively, we systematically explored cell-cell communications and regulons of early thymocytes as well as age-related differences in human thymus by using both bioinformatic and experimental verification, indicating IGFBP5 as a functional marker of thymic involution and providing new insights into the mechanisms of thymus involution.

## 1 Introduction

Thymus is a major lymphoid organ that is essential for building self-tolerance and functional adaptive immune system (1, 2). Thymic epithelial cells (TECs) closely interact with developing thymocytes to provide a suitable environment for lineage induction and thymocyte selection. In turn, developing thymocytes provide essential signals for TECs maturation. The interaction between TECs and thymocytes, known as “thymic crosstalk”, defines the unique ability of the thymic microenvironment to coordinate T cell development (3, 4). However, the organ degenerates shortly after birth termed as age-related thymic involution which is remarkable in human but not in mouse. Age-related thymic involution leads to immune dysfunction in elder humans which is associated with age-related incidences of cancer, infection, and autoimmunity (5). Yet, the process of thymic degeneration has not been well studied due to the lack of thymic involution models. Therefore, it is urgently needed to study the mechanism of human thymus changes with aging.

Thymus seeding progenitors (TSPs) seed at thymus and begin the following T cell specification and commitment in a series of stages that are precisely coordinated by multiple signaling and transcriptional networks (6). For example, NOTCH signaling drives TSP entering a T cell development trajectory and IL7 signaling boosts early T lineage progenitors’ expansion (7, 8). To date, genetic and molecular evidences have identified many of these regulators, but mainly in late stages after T cell commitment and in mouse models (9). There are limited data reporting how immature T cells of human at early stages were regulated. Fortunately, Le et al. provided a high-resolution cellular atlas of the early stages of human immature T cells which motivated us to systematically explore the signaling and regulators of immature T cells by integrating Le et al. data with other available datasets (10). Thymus undergo age-related degenerations with TEC reduction, fibroblast and adipocyte expansion and increasing evidences suggest that thymic involution is mainly caused by thymic stromal cell degeneration, particularly TECs degeneration. For example, gradual loss of FOXN1, which primarily regulates TEC differentiation and homeostasis, has been shown to be associated with age-related thymic involution (11). While, Foxn1-overexpressed embryonic fibroblasts could rejuvenate aged thymic architecture and function in mice (12). However, the mechanistic changes in the aging TECs remain largely unknown. Thanks to Bautista et al. and Park et al. reports which they provided another two high quality and quantity cellular atlas of thymus stroma including TECs (3, 13), we can data-mine the signals and regulators of TECs during aging. The present work aimed to construct a cell atlas of human thymus and study what signals and transcription factors to be involved in aging thymus.

## 2 Materials and methods

### 2.1 Clinical and mouse tissue collection

Human thymic tissues were obtained from patients undergoing cardiac surgery with protocols approved by the Medical Ethics Committee of the Second Affiliated Hospital, Army Medical University following guidelines of the Declaration of Helsinki. Written informed consent was obtained from patients or patient’s guardians. C57BL/6J mice were purchased from Chongqing Tengxin Bio-Technology. Mice experiments were performed according to the guidelines of Laboratory Animal Welfare and Ethics Committee of the Third Military Medical University and the study was carried out in compliance with the ARRIVE guidelines.

### 2.2 Data collection

To obtain single-cell data on human early thymocytes, dataset GSE139042 were acquired from the Gene Expression Omnibus (GEO) database. Dataset GSE147520 was downloaded to obtain enriched human thymic stroma. The dataset downloaded from the Zenodo repository provided most of the thymic immune cells. All data generated or analyzed during this study are freely available in previous publications or in the public domain. All three datasets are raw count matrix. Among them, except for dataset GSE139042, which contains some data from the inDrop platform, the other datasets are all generated by the 10X Genomics platform sequencing. The homogeneity of the datasets was checked to ensure that they can be combined (**Supplementary Table 1**). The sequencing results from 10X Genomics show a high degree of similarity across the board, with only slight variations observed in the data obtained from inDrop. However, we can integrate them using a specialized algorithm.

### 2.3 ScRNA-seq analysis in Python

Single-cell data analysis was performed using Python package Scanpy (version 1.9.1) (14). Quality control and data correction for single-cell samples based on the number of genes detected, the number of molecules detected, and the percentage of mitochondria in each single-cell sample. Cells with fewer than 2000 detected molecules and 500 detected genes were removed from the dataset. Cells with more than 7000 detected genes were considered as potential doublets and removed from the dataset. For the percentage of mitochondria, samples using 10X Genomics platform with more than 10% mitochondria genes were removed, and samples using inDrop platform with more than 20% mitochondrial genes were removed. Scrublet (15) algorithm was applied to calculate scrublet-predicted doublet score to exclude doublets from scRNA-seq data. After data filtration, the concat function was first used to merge data from two different platforms provided by Le et al. and then merged it with data from Park et al. and B et al. to produce a combined dataset. Cell cycle scores were calculated using the score_genes_cell_cycle function. Normalize_per_cell was used to normalize the combined dataset (counts_per_cell_after = 10,000). The top 2,000 high variable genes (HVG) defined by highly_variable_genes were used for principal component analysis (PCA). Cell cycle-dependent changes in gene expression and variation caused by mitochondrial gene expression were regressed out using the regress out function. BBKNN (16), an integration algorithm, was applied to correct batch effects caused by multiple samples and platforms. Clustering was performed using the Leiden algorithm with a resolution of 1.5. Clustering and visualization results were realized by Uniform Approximation and Projection method (UMAP). The rank_genes_groups (method = t-test) function was used to identify markers for each cluster. Cluster cell identity was named by manual annotation using differentially expressed genes and known marker genes.

### 2.4 ScRNA-seq analysis in R

Some sub-clustering was further subdivided using Seurat (version 4.2.0) (17) to achieve a high-resolution annotation. The re-analysis of all subpopulations was performed using similar methods. After extracting the original count matrix of the target subpopulation, we adopted the method of re-dimensionality reduction clustering to obtain more diverse cell types. The same cell filtration conditions as in the Scanpy analysis were taken. The NormalizeData function was used to normalize the data, and the CellCycleScoring function was applied to calculate cell cycle related scores. After the top 2000 HVG were obtained, the ScaleData function was used to remove cell cycle effects and PCA was performed. The RunHarmony function in Harmony (18) was then executed to remove the batch effect and 1-40 PCs were used for UMAP dimensionality reduction. The FindallMarkers function was used to identify markers for each cluster. Cell clusters were annotated by differentially expressed genes and markers that had been reported.

### 2.5 Cell–cell communication analysis

CellChat (version 1.4.0) (19), an algorithm for analyzing the intercellular communication networks at single-cell level, contains ligand-receptor interaction databases for human and mouse. The global intercellular communication network under 19w, 10m and 25y conditions were quantified and compared. To observe trends in cell-cell communication, compareInteractions function was used to compare the total number of interactions and interaction strength or compare them among different cell populations. Comparing the major sources and targets in 2D space to identify the cell populations with obvious changes in sending or receiving signals. To identify the conserved and context-specific signaling pathways, the overall information flow for each signaling pathway were calculated and compared. Those signaling pathways greatly altered were picked up for specific analysis. Comparing the communication probabilities mediated by ligand-receptor pairs from some clusters to other clusters by setting comparison in the function netVisual_bubble.

### 2.6 Gene-regulatory networks

SCENIC (version 1.2.4) (20) is a tool to simultaneously reconstruct gene regulatory networks and identify steady cell states from scRNA-seq data. The pySCENIC package (version 0.11.2) (21), a Python-based implementation of the SCENIC pipeline, has a faster analytical speed. The analysis section was run in pySCENIC. The input to pySCENIC was the scRNA-seq expression matrix. The result was printed in loom file format and imported into SCENIC for visualization. R and Cytoscape were used to visualize the results (22).

### 2.7 Gene ontology (GO) and gene set variation analysis (GSVA)

ClusterProfiler (version 4.2.2) was applied to analyze the enrichment of certain genes (23). Functional enrichment analysis based on the GO database was performed on each cell to explore their possible biological functions. We considered only gene sets with p-value <0.05 were significantly enriched. In addition, 200 hallmark signatures associated with epithelial-mesenchymal transition (EMT) were collected from the molecular signature database (MSigDB) and 1167 genes associated with EMT were downloaded from the Epithelial-Mesenchymal Transition gene database (dbEMT) (24). Gene set variation analysis (GSVA) was performed to calculate the enrichment scores for these TEC clusters.

### 2.8 Immunohistochemical staining

Thymus tissue was fixed in 4% paraformaldehyde (biosharp life science, BL539A), embedded in paraffin, and sectioned to 4 μm thickness. Antigen retrieval was performed by boiling sections in Improved Citrate Antigen Retrieval Solution (Beyotime, P0083). Sections were blocked for 1 hour at room temperature using 5% BSA (Solarbio life sciences, SW3015), followed by incubation with IGFBP5 antibody (Abclonal, A12451) overnight at 4 °C. Staining with HRP Donkey Anti-Rabbit IgG (H+L) (Abclonal, AS038) was performed for 1 h at room temperature. Slides were developed using DAB (Beyotime, P0202) and counterstained with hematoxylin (Beyotime, C0107). All slides were scanned by PreciPoint M8 and analyzed with ViewPoint BETA v 0.8.2.7.

### 2.9 Cell culture and siRNA transfection

Immortalized TECs (iTECs) were cultured in Dulbecco’s modified Eagle’s medium (DMEM, Gibco, USA) supplemented with fetal bovine serum (10%, FBS, Gibco, USA) and Penicillin-Streptomycin Solution (1%, PS, Biosharp, China). Cells were maintained in a humidified incubator with 5% CO_2_ at 37 °C. siRNAs were synthesized by Sangon Biotech (Shanghai, China). siRNAs transfection was carried out by Lipofectamine™ 3000 (Invitrogen™, Lipofectamine™ 3000, USA) according to the manufacturer’s instructions. Total RNA was harvested by RNAiso Plus (Takara, Japan) and protein was collected by RIPA Lysis. Buffer (Beyotime, China) with Phosphatase and Protease Inhibitor Cocktails (Roche, USA) after 48h and 72h, respectively, then stored at -80°C. The siRNA sequences utilized are shown in **Supplementary Table 2**. C57BL/6J mice were sacrificed and thymocytes were purified and co-culture with iTECs cells for 24 h using 24-well Transwell plates (8-μm pore size; Millipore, USA).

### 2.10 Cell proliferation assay and flow cytometry

Cell proliferation was assessed by CCK8 incorporation assay (Beyotime, China) as previously described (25). Single-cell suspensions of thymocytes and iTECs from the experiments were used for flow cytometry with a FACSCanto II (BD Biosciences). The surface staining was performed in FACS buffer, the anti-CD4 (RM4-5), anti-CD8 (53-5.8) were obtained from Biolegend. The staining of Ki67(Invitrogen) was performed with a Cytofix/Cytoperm Fixation/Permeabilization Kit (554714, BD Biosciences) according to the manufacturer’s instructions after surface staining. Data were analyzed by FlowJo (Treestar).

### 2.11 Reverse-transcription quantitative PCR (RT–qPCR)

Total RNA was isolated from iTECs cell with RNAiso Plus via Phenol chloroform extraction. gDNA removing and DNA synthesis were implemented by ABScript III RT Master Mix for qPCR with gDNA remover (ABclonal, China) according to the manufacturer’s instructions. Real-time PCR was performed (Novogene&Kubo, Quantagene q225, China) with SYBR Green qPCR Master Mix (MedChemExpress, China). The relative mRNA level was calculated by the 2−ΔΔCt method and normalized to β-actin. RT–PCR primers were synthesized by Sangon Biotech (Shanghai, China) and listed in **Supplementary Table 3**.

### 2.12 Western blot

Total protein was separated by 4-20% Tris-HCl/SDS-polyacrylamide gels (Beyotime, BeyoGel™ Plus PAGE, China) and transferred to PVDF membranes. Then, the membranes were incubated with blocking buffer (Beyotime, QuickBlock™ Western, China) for 1h, with primary antibodies (IGFBP5 (1:1000, A12451, ABclonal), β-actin (1:5000, ab8226, Abcam)) overnight at 4 °C and with secondary antibody (1:5000, HRP Donkey Anti-Rabbit IgG (H+L) (AS038), ABclonal) for 1h at room temperature. Membranes were imaged by ChemiDoc™ Touch (ChemiDoc Touch 1708370, Bio-red, USA).

### 2.13 Statistical analysis

Statistical analysis and graphics were generated using GraphPad Prism version 5.0 (GraphPad Software, La Jolla, CA, USA; www.graphpad.com). All statistical analyses were performed using a paired two-sided t test. A difference was considered to be statistically significant at *P< 0.05, **P< 0.01 and ***P< 0.001, and not significant (NS) when P> 0.05.

## 3 Results

### 3.1 Integration of cell atlas of human thymus

**Figure 1:**
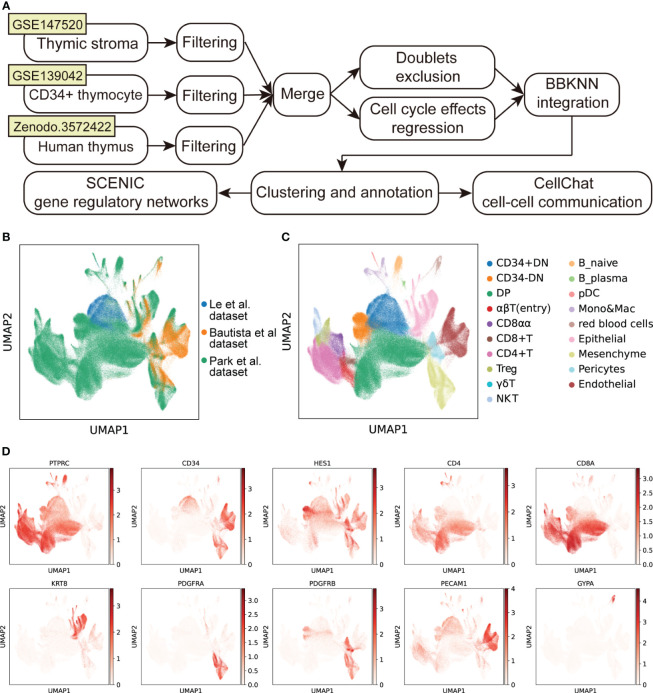
Integration of scRNA-seq datasets of human thymus. **(A)** Schematic flowchart showing the processing, integration, and analysis of the cell atlas of human thymus. **(B)** UMAP visualization of human thymus colored by data source. **(C)** UMAP visualization of the cellular composition of human thymus colored by cell type (DN, double-negative T cells; DP, double-positive T cells; pDC, plasmacytoid dendritic cells; Mono, monocyte; Mac, macrophage). **(D)** UMAP visualization of the expression of well-known marker genes used for cell cluster identification.

To gain insight into the mechanisms of development and degeneration of thymus at different ages, we downloaded three recently published scRNA-seq datasets of human thymus to generate an integrated cell atlas (3, 10, 13) (**Figure 1A**). After strict quality control, a total of 350,678 cells from 36 samples were retained for downstream analysis. Cell cycle effects were viewed and regressed out. BBKNN was used to exclude the batch effects across the combined dataset (**Figure S1A**) and datasets were well integrated (**Figure 1B**). After clustering and visualization, we annotated the dataset into 19 different cell types (**Figure 1C**), which can be clearly identified by specific marker genes (**Figure 1D**; **Supplementary Figure 1B**). We divided differentiating T cells into CD34^+^ double negative (CD34^+^ DN), CD34^-^ double negative (CD34^-^ DN), double positive (DP), αβT (entry), CD8^+^ single positive (CD8^+^ T), CD4^+^ single positive (CD4^+^ T), CD8αα^+^, FOXP3^+^ regulatory (Treg) and γδT cells. We also identified other immune cells including natural killer (NK) cells, B cells, macrophages & monocytes (Mono&Mac), and DCs. Finally, the integrated human thymus atlas benefited us for the following analysis.

### 3.2 Cell–Cell communication of early thymocytes

**Figure 2:**
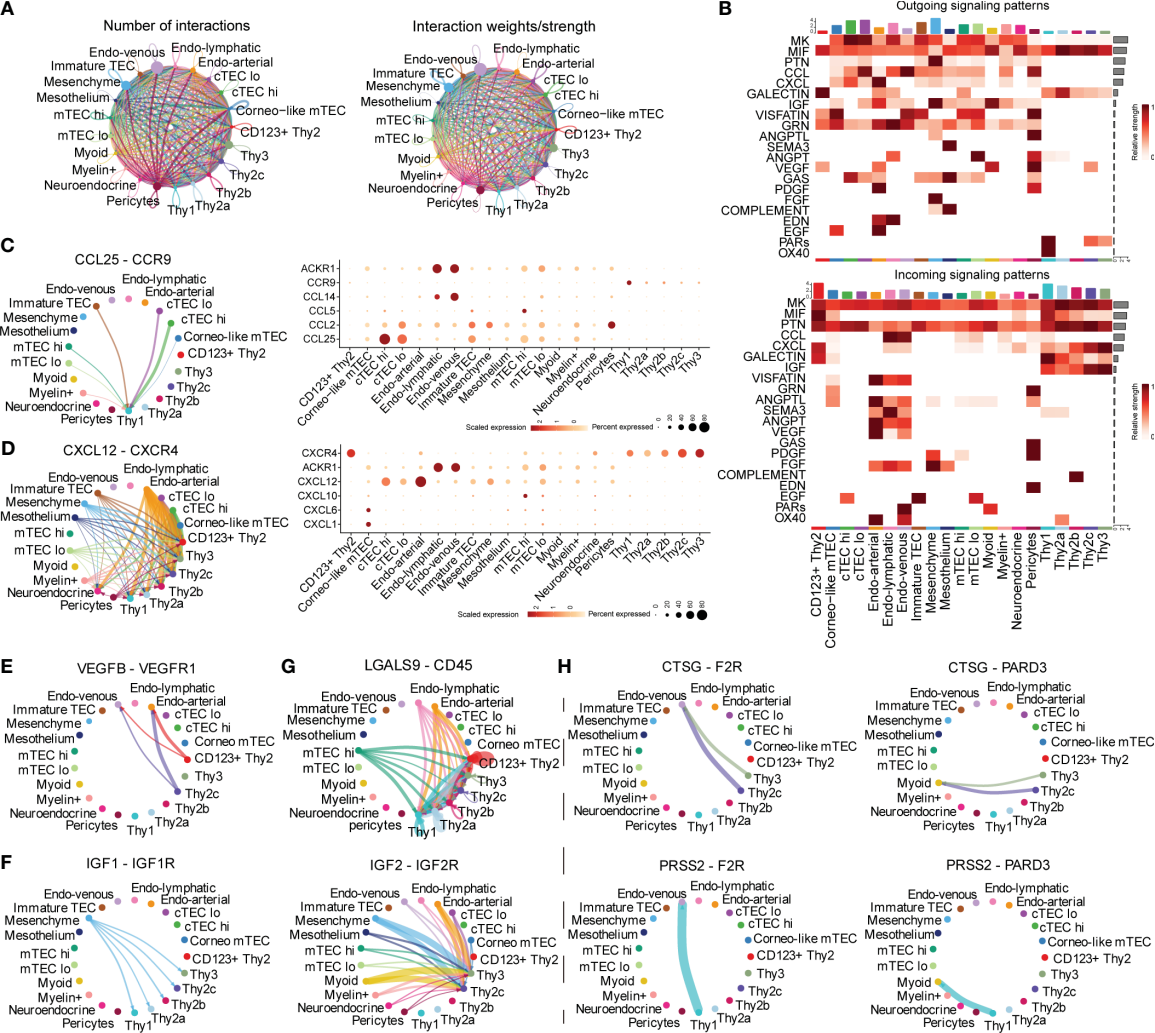
Inference of Cell–Cell communications by CellChat of early thymocytes. **(A)** Circle plots of the interaction quantity and interaction strength among cell types. Here and in later figures, dot represents a cell type, line represents the interaction, and color encodes the number of ligand-receptor pairs **(B)** Heatmaps of outgoing and incoming signaling flows of each cell population mediated by individual signaling axes. Here and in later figures, color represents maximum normalized mean strength. **(C, D)** Circle plot show cell–cell communication mediated by CCL/CXCL (Left panel). Dot plot of ligand receptor pair expression of CCL/CXCL (Right panel). **(E)** Circle plots show cell–cell communication mediated by VEGF **(E)**, GALECTIN **(F)**, IGF **(G)**, PARs **(H)**.

A systematical overview of the intercellular communications of human early thymocytes remained incomplete and we thus investigated intercellular signaling to resolve what and how the signaling impacted development of early thymocytes. CD34^+^ early thymocytes, TECs and thymic stroma were enriched and analyzed (**Supplementary Figure 2A**). We annotated TECs subcluster as cTEC^hi^, cTEC^lo^, immature TEC, mTEC^lo^, mTEC^hi^, corneocyte-like mTECs, neuroendocrine, muscle-like myoid, and myelin^+^ epithelial cells. The immune cells were further divided into Thy1, CD123^+^ Thy2, Thy2a, Thy2b, Thy2c and Thy3 using accepted markers (3, 10, 26). CellChat was performed to predict the ligand-receptor pairs among early thymocytes and other cells (**Supplementary Figure 2B**; **Supplementary Table 4**). The number and strength of interactions were shown, indicating that there was extensive “thymic crosstalk” not only between TECs and thymocyte but also between stroma cells and thymocytes (**Figure 2A**). Ligand-receptor pairs were then divided into 21 signaling pathways. Among them, MK, MIF, PTN, CXCL and GALECTIN signaling were mainly involved in the incoming signaling pattern between early thymocytes and other cells (**Figure 2B**, bottom panel), while the outgoing signaling pattern mainly included MIF and PARs signaling (**Figure 2B**, top panel).

We further characterized and visualized specific ligand-receptor pairs. Chemokines were reported to be essential for early thymocytes seeding and migration in thymus and our following analysis was in accordance with these reports (27). CCL25-CCR9 was illustrated to communicate between TECs and Thy1 (**Figure 2C**). While, CXCL12 is emitted by nonimmune cells (mainly stroma cells) and received by early thymocytes (**Figure 2D**, Left panel). As early thymocytes developed, the interaction mediated by this signaling was stronger and CXCR4 expression was also higher (**Figure 2D**, Right panel). These, together with previous literature descriptions, suggested that CCL25-CCR9 and CXCL12-CXCR4 signaling were involved in early thymocytes migration and development (28). Thymocyte proliferation is also important for thymus maturation, we found VEGF/IGF signals were involved. Specifically, VEGF signal emitting from Thy2 cells (identified as DN2 in mice) may facilitate the growth of endothelial cells (**Figure 2E**). IGF signaling existed between mesenchymal cells and early thymocytes except CD123^+^ Thy2 indicating early T cells also needs IGF proliferating signaling consistent with other’s reports in mouse models (**Figure 2F**) (27, 29). LGALS9 was reported to regulate apoptosis of thymocyte and our analysis revealed that most endothelial cells and mTEC^hi^ expressed the ligand of galectin-9 (LGALS9) and all early thymocytes expressed its receptor (**Figure 2G**) (30). On the other side, thymocytes can also emit signals such as the proteases, including Cathepsin G (CTSG) and PRSS2 (**Figure 2H**). CTSG is an endo-protease that plays an important role in regulating chemotaxis with the ability to degrade CXCL12 (31). So, CTSG and PRSS2 may also affect the migration of Thy2c and Thy3 cells. In short, our analysis revealed that the TECs and stroma cells provided various signals for early thymocyte migration, proliferation, and apoptosis.

### 3.3 Transcriptional regulatory networks of early thymocytes

**Figure 3:**
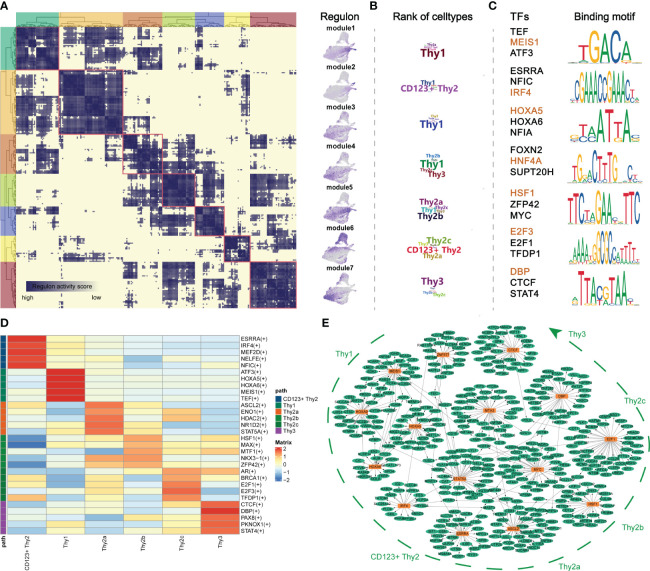
Transcriptional regulatory networks of early thymocytes. **(A)** Identification of regulon modules using SCENIC. Heatmap shows the similarity of different regulons based on the AUCell score (Left panel). UMAPs illustrate the average AUCell score distribution for different regulon modules (Right panel). **(B)** Wordcloud plots showing enrichment of celltypes in regulon modules. **(C)** Representative TFs and corresponding TF binding motifs in different regulon modules. **(D)** Heatmap showing TFs enriched in different celltypes. The level of regulon expression is represented by color depth. **(E)** Integrated gene-regulatory networks of the regulons. The symbol "(+)" means that the Spearman correlation between the TF and the potential target is positive (positive-correlated targets).

The development of lymphocytes was precisely regulated by critical transcriptional factors (TF) (8). To obtain an accurate process of early thymocyte specification, we analyzed CD34^+^ thymocytes exclusively (**Supplementary Figures 3A, B**). To systematically predict cell-type-specific transcriptional regulatory networks, we identified TF regulators based on co-expression and motif enrichment by using SCENIC (21). Regulon modules were identified based on the Connection Specificity Index (CSI) (32). We identified seven TF regulon modules that were active in a cell-type-specific manner (**Figures 3A, B**). Then, representative TF regulons across different cell types were presented based on regulon activity scores (**Figure 3C**). MEIS1 and HOXA5 were enriched in Thy1 cells and may maintain the stemness of T precursor cells as the upregulation of them were reported in acute lymphocytic leukemias (33). IRF4 was highly expressed in module 2 corresponding to CD123^+^ Thy2 and may regulate the fate choice between T progenitor and myeloid lineage in accordance with previous reports (34). For CD123^-^ Thy2 cells, the representative TFs HSF1, MYC and E2F family proteins, acted on mitosis, cell cycle regulation and DNA replication, and they may collaboratively promote cell proliferation of Thy2 which is needed for the following T cell β-selection (35). Interestingly, we noticed that the CTCF, a critical chromatin organizer, was presented in Thy3 cells and we speculated that a chromatin barrier is establishing in Thy3 cells to lock cell fate into the T lineages (36). Besides, we also identified cell-type-specific TFs according to their expression level as shown in **Figure 3D**. And these cell-type-specific TFs were highly consistent to the TF regulons in the 7 modules above. Furthermore, the representative TF regulons and their associated target genes were organized into gene regulatory networks with cell differentiation trajectory (**Figure 3E**). In summary, we resolved major TFs and transcriptional regulatory network at different stages of early T cells development.

### 3.4 Alterations in Cell–Cell Communications in TECs at different ages

**Figure 4:**
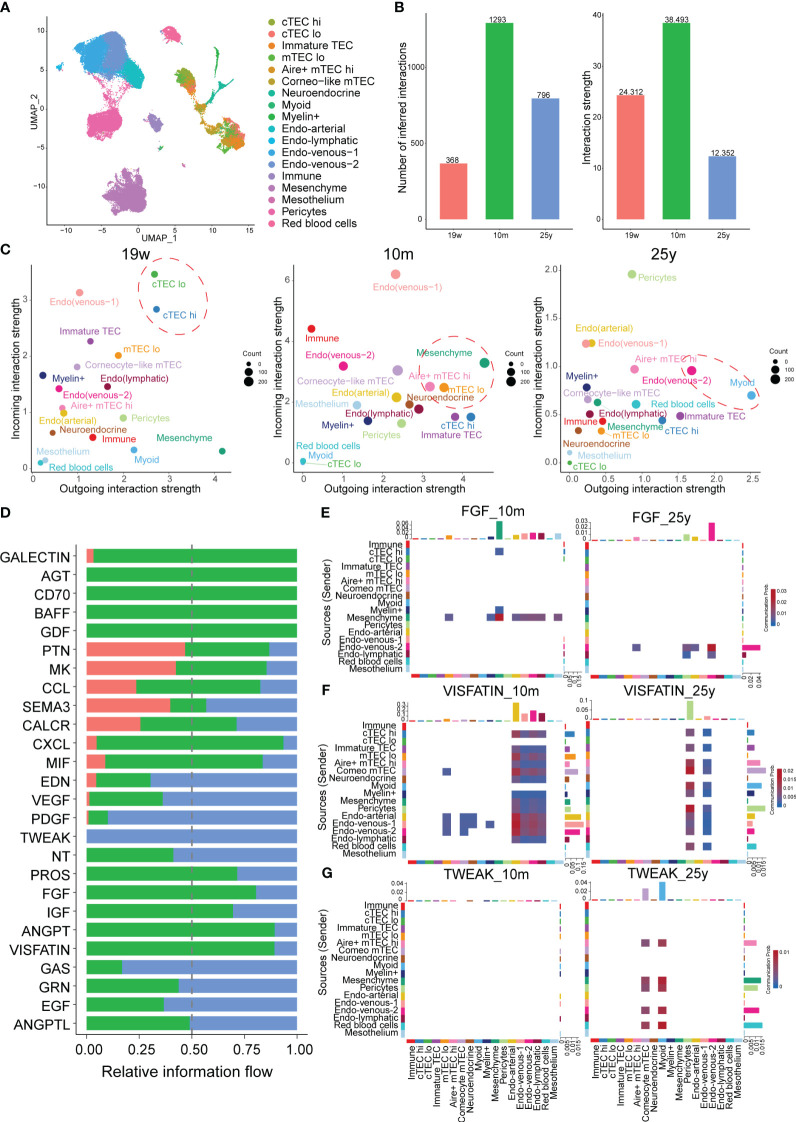
Alterations in Cell–Cell Communications in TECs at different ages. **(A)** UMAP visualization of thymic stroma colored by cell types. **(B)** Bar plots of the interaction quantity and interaction strength among different age groups. (Thymus with 19-week, 10-month and 25-year) **(C)** Scatter plots compare the outgoing and incoming interaction strength in 2D space, color represents maximum normalized strength. **(D)** Bar plots of the ranking of signaling axes by overall information flow differences among different age groups. Red-colored labels, 19w; green-colored, 10m; blue-colored, 25y. Heatmaps show outgoing and incoming of FGF signaling **(E)**, VISFATIN **(F)**, TWEAK **(G)** associated with each cell population at different ages.

Signaling from degenerated TECs may initiate thymic involution (2). Therefore, we further focused on TECs from different age groups using the CellChat package to gain insight into the signaling changes during thymic involution (**Figure 4A**; **Supplementary Figure 4A**). On one side, the total number and strength of cell-cell communication from different ages were illustrated and there were more and stronger signaling after postnatal compared to prenatal stage, while with aging in the adult stage, there was a dramatic decrease in both the number and strength of signaling compared to the postnatal stage (**Figure 4B**). On the other side, by comparing the outgoing and incoming signaling strength between different ages, we discovered that the signaling pattern significantly changed with aging. In detail, mature mesenchyme, mTECs including Aire^+^ mTEC^hi^, Neuroendocrine mTECs dominated the incoming and outgoing signaling in postnatal thymus compared to the prenatal thymus while the signaling path switched to the endothelial cells, Myoid mTECs in adult thymus (**Figure 4C**). Furthermore, information flow analysis indicated that signaling contents were also significantly changed among the prenatal, postnatal and adult thymus (**Figure 4D**). For example, FGF signaling (FGF7:FGFR1), produced by mesenchymal cells and involved in the proliferation and differentiation of TECs, was reduced in adult thymus (**Figure 4E**) (27). And GALECTIN signaling (LGALS9:CD45), above analysis of LGALS9 acting on endothelial cells and early thymocytes, wasn’t detected in adult thymus (**Supplementary Figure 4B**). Notably, ANGPT, CXCL and VISFATIN (NAMPT) signaling were drastically decreased in adult thymus (**Figure 4F**; **Supplementary Figures 4C, D**). In contrast, TWEAK (TNFSF12:TNFRSF12A) signaling was acting in only adult thymus (**Figure 4G**). Taken above analysis together, our data suggested that critical signals for thymic function were altered to varied degrees in aging thymus.

### 3.5 Alterations in gene regulatory networks in TECs with aging

**Figure 5:**
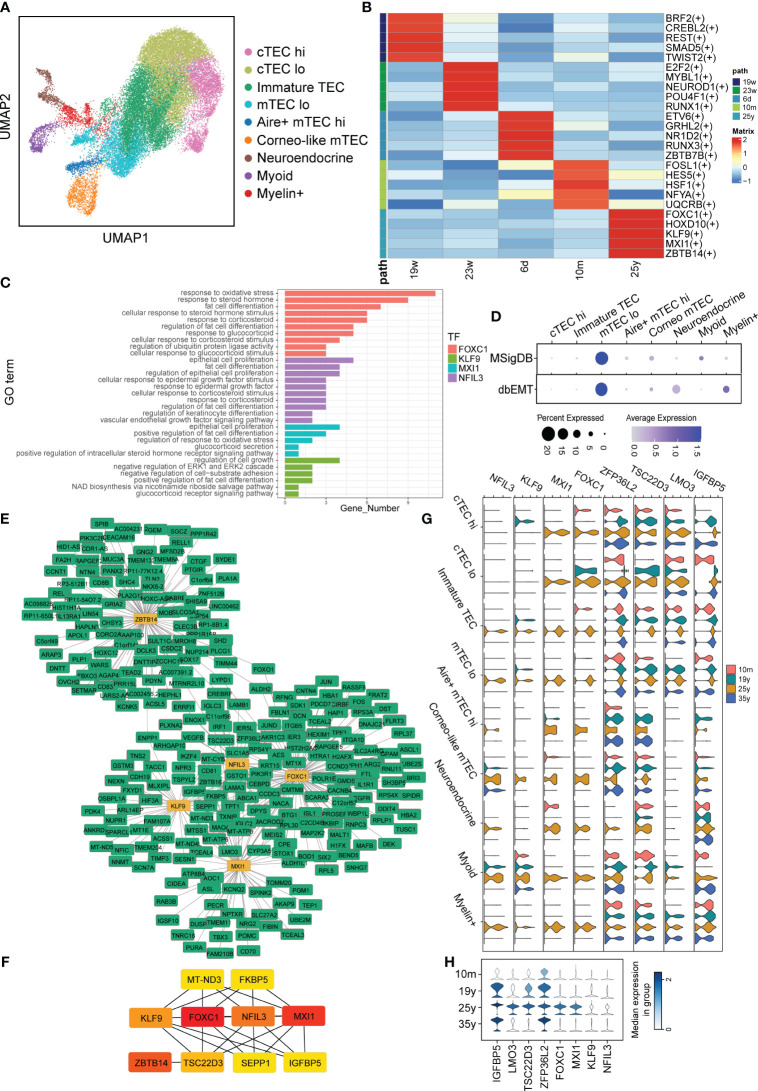
Alterations in Gene Regulatory Networks in TECs with aging. **(A)** UMAP visualization of TECs colored by cell types. **(B)** Heatmap showing TFs enriched in TECs with aging. The level of RSS is represented by color depth. **(C)** GO analysis of regulons of aging TECs. **(D)** Dot plots of enrichment of EMT-associated gene sets from MSigDB (Top panel) and dbEMT (Bottom panel) in aging TECs by GSVA. **(E, F)** Integrated gene-regulatory networks of the regulons in aging TECs. **(G)** Violin plots of regulon (IGFBP5, TSC22D3, LMO3, ZFP36L2, NFIL3, KLF9, MXI1 and FOXC1) expression in different TECs subset. **(H)** Violin plots of genes (IGFBP5, TSC22D3, LMO3, ZFP36L2) expression in all TECs with aging. The symbol "(+)" means that the Spearman correlation between the TF and the potential target is positive (positive-correlated targets).

Considering that signaling pattern of TECs were significantly impaired during aging, we therefore further analyzed the regulon changes of TECs with aging. TECs were extracted for following analysis (**Figure 5A**). We performed GRN inference using computational algorithms implemented in pySCENIC and 443 regulators involving a total of 14,428 genes were chosen for further exploration. We next defined a regulator specificity score (RSS) for each regulon in each cell type based on activity scores (37) (**Supplementary Table 5**) and determined regulons with the highest RSS values as primary regulators for corresponding aging identity. Notably, our RSS analysis revealed that the most specific regulons of aging thymus TECs included Forkhead box C1 (FOXC1), KLF9 (Kruppel Like Factor 9), MXI1 (MAX Interactor 1), NFIL3 (Nuclear Factor, Interleukin 3 Regulated) and ZBTB14 (Zinc Finger and BTB Domain Containing 14) (**Figure 5B**). FOXC1 induced EMT reported in epithelial tumor cells and suppression of FOXC1 can partially reverse the EMT process (38). While, knockdown of KLF9 in zebrafish embryos impaired T lymphopoiesis, MXI1 were reported to correlate with cell proliferation and migration and NFIL3 has been reported as a glucocorticoid-regulated gene (39–41). To further specify the roles of regulons of aging TECs, we performed Gene Ontology (GO) enrichment, gene set variation analysis (GSVA) and cytoscape analysis. In accordance, the enriched GO pathways mainly included fat cell differentiation and response to steroid, glucocorticoid, corticosteroid and EGF/VEGF signaling (**Figure 5C**). Transdifferentiation of TECs into adipocytes with age in mice has been reported and a recent study indicated that EMT process in TECs were through the CD147/p-Smad2/FoxC2 signaling pathway in mouse models (42). We further surveyed whether EMT involved in human TECs. Indeed, GSVA examined that EMT process was enriched in aging TECs and mTEC^lo^ subpopulation had the highest correlation, indicating that mTEC^lo^ subpopulation may initiate the dedifferentiation of TECs to adipocytes (**Figure 5D**). Interestingly, cytoscape analysis indicated IGFBP5 (insulin-like growth factor binding protein 5) was their main target of the above regulons (**Figures 5E, F**). Furthermore, the expression levels of FOXC1, KLF9, MXI1 and their target IGFBP5 were significantly increased in a variety of adult TEC subclusters (**Figure 5G**). But only IGFBP5 displayed gradually increased-to-highest expression in elder (35y) TECs (**Figure 5H**). This is consistent with what was reported in Bautista et al. ‘s study. IGFBP5 has also been reported to increase cell invasion and inhibit cell proliferation by EMT in the human glioma tissues (43). Taken the above analysis data together, we thus speculated that upregulated regulons of aging TECs promoted thymic involution via EMT process.

### 3.6 IGFBP5 is a maker of thymic involution

**Figure 6:**
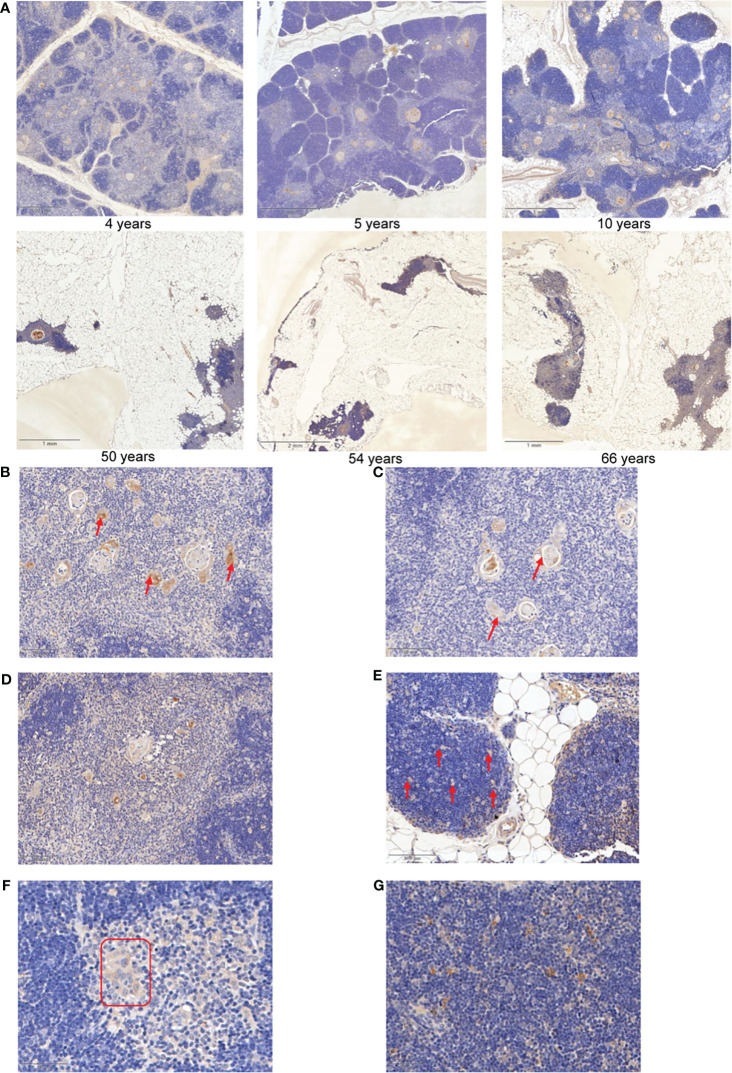
IGFBP5 is a maker of thymic involution. **(A)** Representative IHC images of IGFBP5 at different ages in human thymus. **(B, C)** Representative IHC staining showing IGFBP5 in existed and forming Hassall’s corpuscle. **(D)** IHC images showing IGFBP5 at peri- Hassall’s corpuscle. **(E)** IGFBP5 staining in cortex of human thymus. **(F, G)** Staining of IGFBP5 in cortex and medulla of mouse thymus. The position indicated by the red arrows and red triangle is where IGFBP5 staining is evident.

Our above analysis provided hints that IGFBP5, the target gene of critical TFs, may be a marker correlating to thymic involution. To validate whether IGFBP5 increases with aging in thymic tissue, clinical samples with a wide range of ages (4/5/10/15/18/44/50/54/59/66) were collected for IHC staining (**Supplementary Table 6**). The IHC images confirmed that aged thymus displays obvious TEC reduction, fibroblast/adipocyte expansion and strong IGFBP5 staining (**Figure 6A**). Surprisingly, we also noticed that a strong anti-IGFBP5 positive staining appeared in Hassall’s corpuscle, which was used to be regarded as a microstructure deriving from involuted TECs and associated with ageing of thymus (44). IGFBP5 staining was also captured in forming and fusing Hassall’s corpuscle (**Figures 6B, C**). Besides, mTECs adjacent to Hassall’s corpuscle also showed strong staining of IGFBP5, forming a satellite-like microstructure in thymus (**Figure 6D**). Besides, there is also a moderate staining on cortical thymic epithelial cells (**Figure 6E**). Interestingly, similar feature could be also found in aging c57BL/6 mouse thymus (**Figures 6F, G**). Thus, the IHC result was perfectly identical to mRNA expression pattern of single-cell data, implying that potentially predictive and functional value of IGFBP5 in aging thymus.

### 3.7 IGFBP5 inhibits thymocyte proliferation-related genes expression

**Figure 7:**
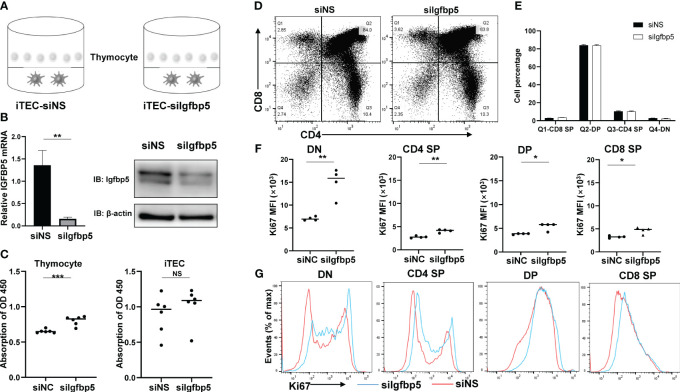
IGFBP5 inhibits thymocyte proliferation-related genes expression. **(A)** Co-culture system of IGFBP5 knockdown iTEC cell line and mouse thymocytes. **(B)** IGFBP5 knockdown efficiency in iTECs. Left panel, mRNA expression of IGFBP5 was detected by RT-qPCR. Right panel, the protein level of IGFBP5 detected by WB. **(C)** CCK8 assays detected cell proliferation in thymocytes and iTECs (***P< 0.001). NS is not significant. **(D)** Percentage of CD8^+^SP, DP, CD4^+^SP, DN via Flow cytometry visualized by dot plot and statistic result **(E)**. **(F)** Expression level of Ki67 in DN, CD4^+^SP, DP, CD8^+^SP. MFI, mean fluorescence intensity (*P< 0.05 and **P< 0.01). **(G)** Ki67 positive events in DN, CD4^+^SP, DP, CD8^+^SP cells.

To verify the role of IGFBP5 in thymus, we transfected small interfering RNA targeting at IGFBP5 (siIGFBP5) or non-specific mock (siNS) in mouse iTEC lines and co-cultured them with primary mouse thymocytes (**Figure 7A**). To firstly quantify the effect of knockdown, we examined relative IGFBP5 mRNA levels by RT-qPCR and protein level by Western blot, and the results showed that IGFBP5 indeed decreased significantly (**Figure 7B**). We then tested cell proliferation rate of thymocytes and iTECs in siIGFBP5 and siNS groups, and CCK8 results showed that proliferation rate of iTECs didn’t change, while, the silencing of IGFBP5 markedly and significantly promoted proliferation of thymocytes (**Figure 7C**). To resolve which cell type of thymocytes were affected by knockdown of IGFBP5, we further used flow cytometry to distinguish thymocytes into DN (CD4^-^CD8^-^), CD4^+^SP (CD4^+^CD8^-^), CD8^+^SP (CD4^-^CD8^+^) and DP (CD4^+^CD8^+^) (**Figure 7D**). The number of thymocytes subsets changed little between the two groups (**Figure 7E**). Consistent with CCK8 assay, the proliferation levels of all of thymocytes subsets, indicated by Ki67 MFI level, increased in siIGFBP5 group (**Figure 7F**). But Ki67 positive percentage of DN and CD4^+^SP cells were mostly increased while DP and CD4^+^SP cells were comparable between siIGFBP5 and siNS group (**Figure 7G**). We speculated that IGF receptors may only be expressed in DN and CD4^+^SP cells. We thus examined the expression level of IGF family and its receptors using our single-cell dataset. In accordance, IGF1R and IGF2R expressions in DN cells were significantly higher than others (**Supplementary Figure 5**). In summary, our results suggested that IGFBP5 derived from TECs can effectively inhibit thymocytes proliferation-related genes expression via IGF signaling.

## 4 Discussion

Thymus is one of the first organs to experience age-related functional decline during which TECs is diminished and fat tissue replace thymus stroma, together resulting in the decline of mature T cell output. However, limited information of this process are available and effective interventions to rejuvenate thymus functions are currently lacking (27). Single-cell analysis technologies have improved our understanding of the cellular compositions and functions in a systematic manner. Here, we data-mined published scRNA datasets of human thymus, uncovered critical regulators of both early thymocyte and TECs, and provided new target and mechanism of thymus involution.

Seeding and developing of TSPs are dependent on thymic stroma which provides signaling. We found chemokines including CCL25/CXCL12 and proteases including CTSG/PRSS2 play roles in early thymocyte homing and migration. Interestingly, Žuklys et al. previously reported that direct targets of Foxn1 in TECs (by chromatin immunoprecipitation sequencing) are chemokines and proteases, including CCL25, CXCL12 and PRSS16 (45) consistent with our founding. Besides, our results also revealed that CCL25/PRSS2 signals were received by only Thy1 cells, while, CXCL12/CTSG signals by Thy1-3 cells. We speculated that CCL25/PRSS2 support the recruitment of thymocyte progenitors while CXCL12/CTSG provide directional cues for migration of thymocytes. In human, transcriptional events of early thymocytes are largely unknown because of their small numbers and therefore, we also inferred a trajectory from Thy1 to Thy3. Our analysis indicated that, consistent with their murine counterparts of DN1-3 cells, Thy1 cells maintain multilineage differentiation ability, CD123^+^ Thy2 cells were potential for myeloid lineage, CD123^-^ Thy2 cells were expanding and Thy3 cells finally commit to T lineage (36). However, further biochemistry and genetic study is needed to clarify the detailed development of human early thymocytes.

Signaling path and contents are substantially altered in the adult thymus compared to early life. TECs couldn’t provide enough signals and instead endothelial cells dominate the signaling pattern in aging thymus. This is probably due to functional TECs loss and vascular remodeling in aging thymus. Besides, critical signals for normal thymic function such as FGF, GALECTIN, ANGPT, CXCL and VISFATIN signaling decrease in aging thymus compared to early life. Instead, TWEAK signaling is increasing in the adult thymus and studies have demonstrated that TWEAK can promote EMT in human bronchial epithelial cells (46). We assumed that TWEAK is either essential or partially involved in EMT of aging thymus. Indeed, EMT of TECs during thymic degeneration has been increasingly approved (42). Our analysis indicated that aging TECs enriched EMT pathway and the identified regulons (FOXC1, KLF9, MXI1, NFIL3, IGFBP5) of aging TECs were reported to be involved in EMT process of other tissues (47, 48). Thus, these results together suggest that TECs undergo EMT during age-related involution which can be impacted by several important regulators.

Our data verified that IGFBP5 is significantly upregulated at TECs and Hassall’s corpuscle in the aging thymus, and the expression of proliferation-related genes in thymocytes was significantly different when IGFBP5 was knocked down or not. However, there was no significant change in the number of thymocytes. IGFBP proteins are secreted into microenvironment and known to have the ability to form complexes with IGF (49). Consistent with this notion, our data showed that downregulation of IGFBP5 in thymocyte-TEC co-culture system promoted the expression of proliferation-related genes in thymocytes, especially in the DN cells. And DN cells expressed the highest level of IGF receptors. We speculated that in the aging thymus, IGFBP5 deprived from aging TECs may bind most of the IGF and function as the IGF sponges, resulting in the lack of IGF growth signals to thymocytes, which in turn degenerates the TECs by “thymic crosstalk” (50). At present, we conclude that IGFBP5 can be used as a significant marker indicating the degree of thymic involution and may have a function in regulating thymocyte proliferation. However, further study is needed to validate its function of the regulation of thymus involution *in vivo*.

Collectively, we report here data to show important factors including IGFBP5 that induce EMT of TECs and adipogenesis processes in thymic involution. These factors may provide potential targets for preventing age-related thymic regression and a framework for optimizing thymus reconstitution in T-cell-deficient patients.

## Data availability statement

The original contributions presented in the study are included in the article/**Supplementary Material**. Further inquiries can be directed to the corresponding author.

## Ethics statement

The studies involving humans were approved by Medical Ethics Committee of the Second Affiliated Hospital, Army Medical University. The studies were conducted in accordance with the local legislation and institutional requirements. Written informed consent for participation in this study was provided by the participants’ legal guardians/next of kin. The animal study was approved by Laboratory Animal Welfare and Ethics Committee of the Third Military Medical University. The study was conducted in accordance with the local legislation and institutional requirements.

## Author contributions

XY: Investigation, Software, Writing – original draft. XC: Methodology, Visualization, Writing – original draft. WW: Investigation, Project administration, Validation, Writing – review & editing. SQ: Funding acquisition, Investigation, Writing – review & editing. BL: Data curation, Formal analysis, Writing – original draft. JZ: Funding acquisition, Resources, Writing – review & editing. JC: Investigation, Project administration, Writing – review & editing. CH: Project administration, Writing – review & editing. YT: Investigation, Writing – review & editing. YX: Investigation, Writing – review & editing. WG: Supervision, Writing – review & editing. YW: Supervision, Writing – review & editing.
