# Supplementary material for: Transcriptional profile of human thymus reveals IGFBP5 is correlated with age-related thymic involution
Source: Front Immunol. 2024 Jan 22;15:1322214. doi: 10.3389/fimmu.2024.1322214 (PMC10839013; doi:10.3389/fimmu.2024.1322214)
Supplement: Supplementary file 7 [file DataSheet_1.docx]

Supplementary Material

**Identification of IGFBP5 as regulators in human age-related thymic involution by scRNA-seq dataset**

**Xiaojing Yang^1^****^†^, Xichan Chen^2†^, Wei Wang^3^, Siming Qu^4^, Binbin Lai^5^, Ji Zhang^2^, Jian Chen^2^, Chao Han^2^, Yi Tian^2^, Yingbin Xiao^3^, Weiwu Gao^2*^, Yuzhang Wu^1,2*^**

*** Correspondence:**

Weiwu Gao: gaoweiwu123@hotmail.com

Yuzhang Wu: wuyuzhang@iiicq.vip

# Supplementary Figures


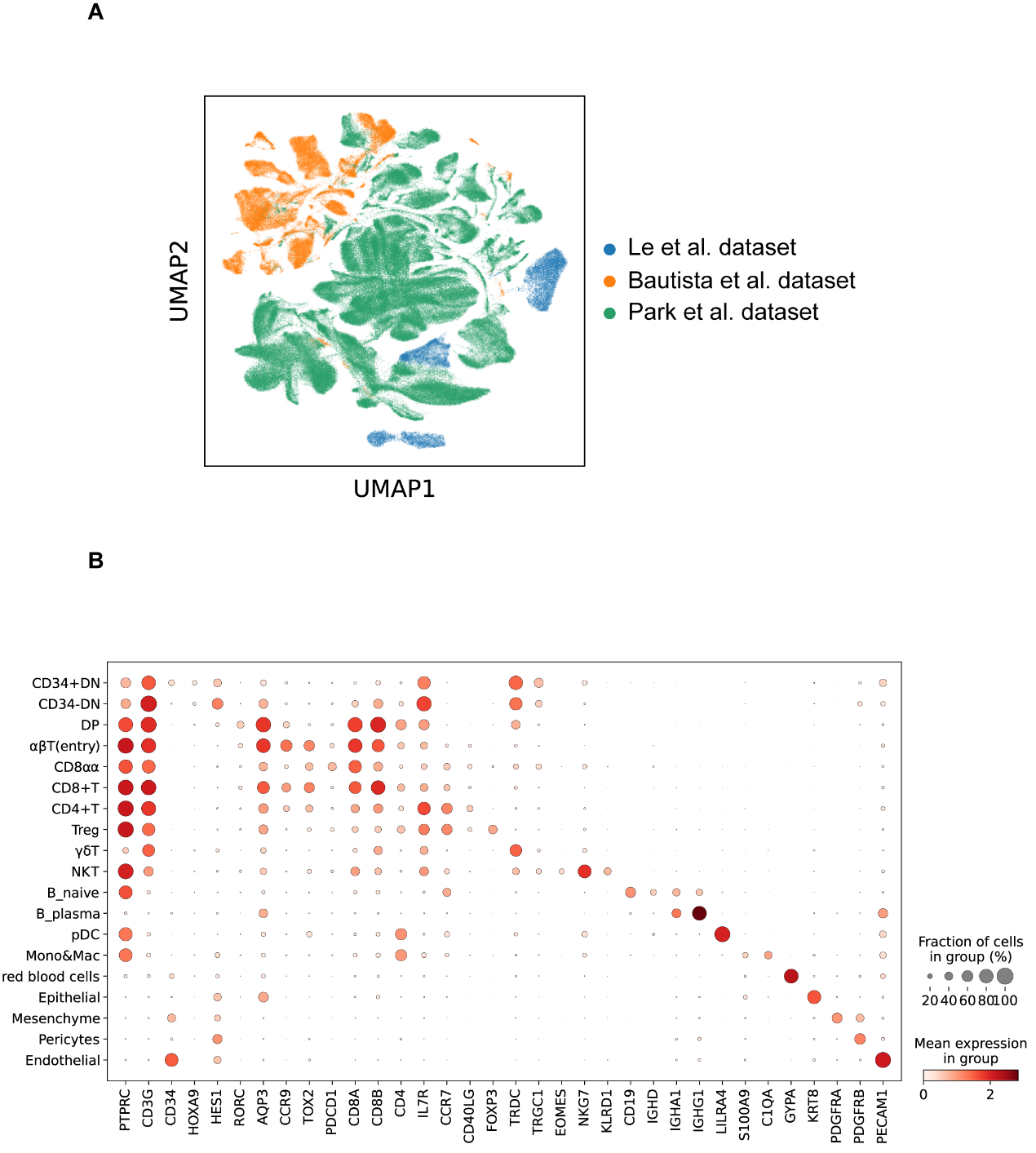


**Supplementary Figure 1.** **(A)** UMAP visualization of the entire dataset before batch alignment. Cells are colored by data source. **(B)** Dot plot showing marker gene expression for annotated cell types. Color represents maximum-normalised mean expression of marker genes in each cell group, and size indicates the proportion of cells expressing marker gene.


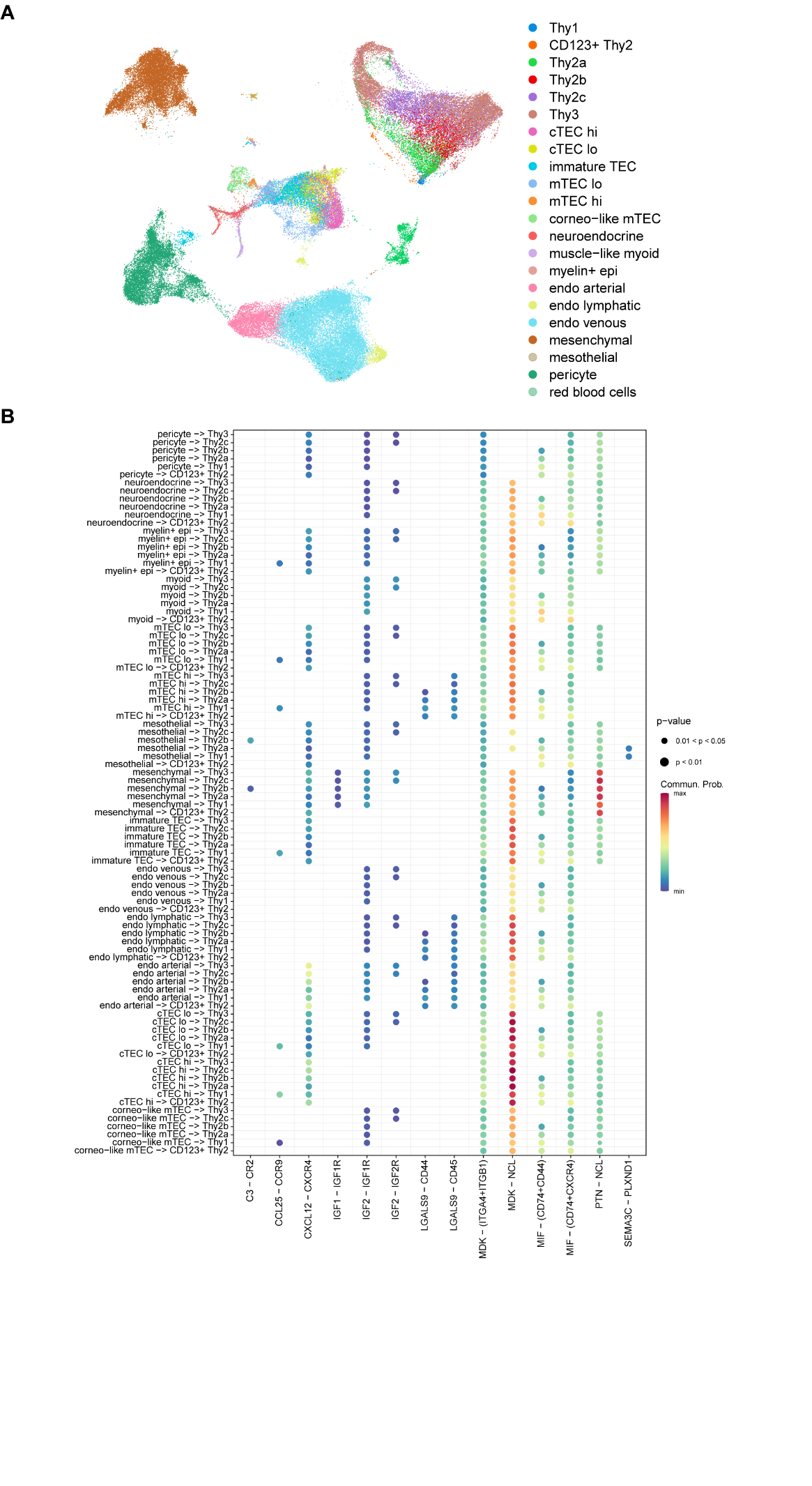


**Supplementary Figure 2.** **(A)** UMAP visualization of the cellular composition of the human thymocytes and thymic stroma colored by cell type. **(B)** Dot plot showing cell type specific expression of signaling pathways.


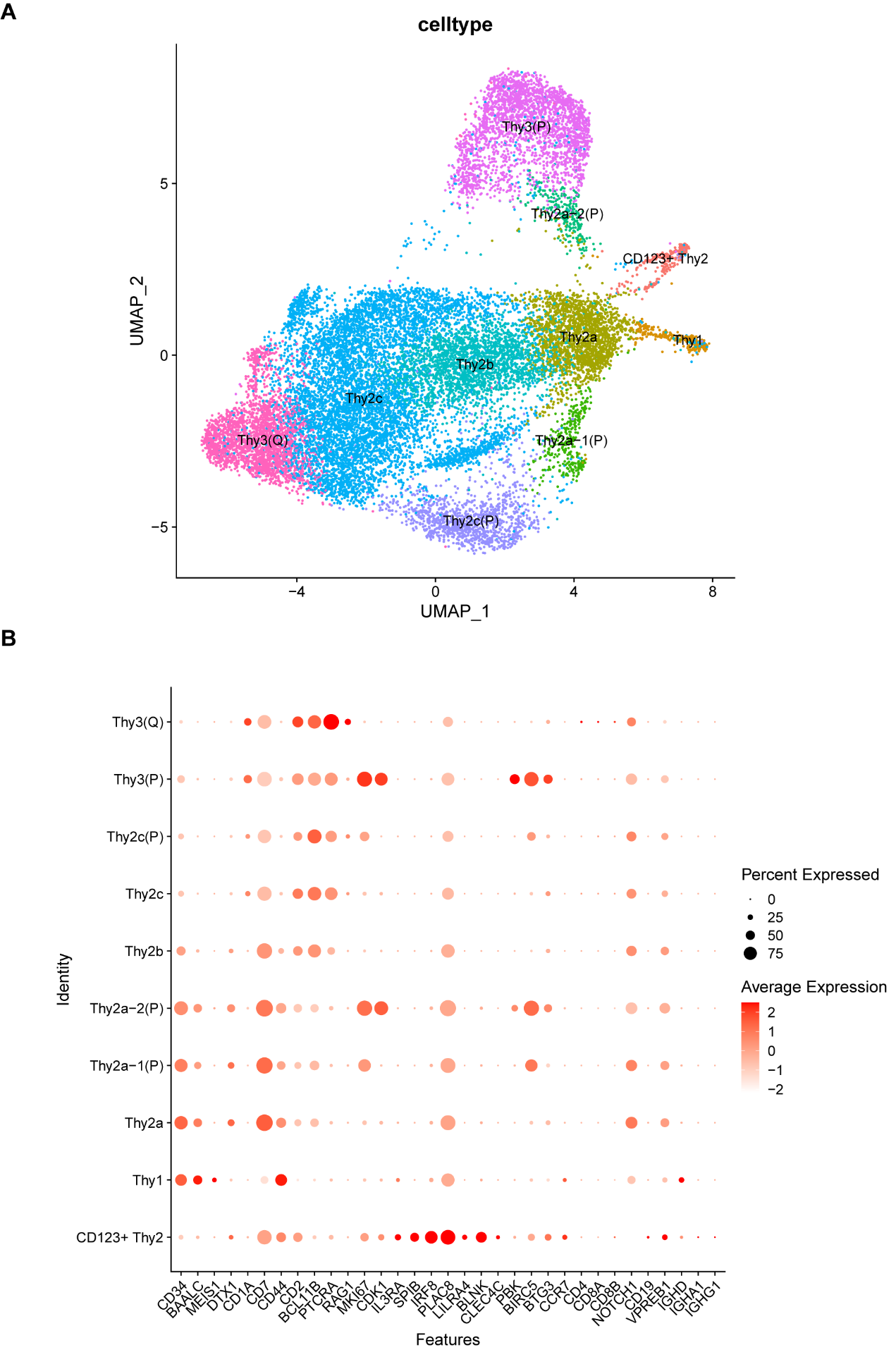


**Supplementary Figure 3.** **(A)** UMAP visualization of the cellular composition of CD34^+^ early thymocytes colored by cell type. **(B)** Dot plot showing marker gene expression for annotated cell types.


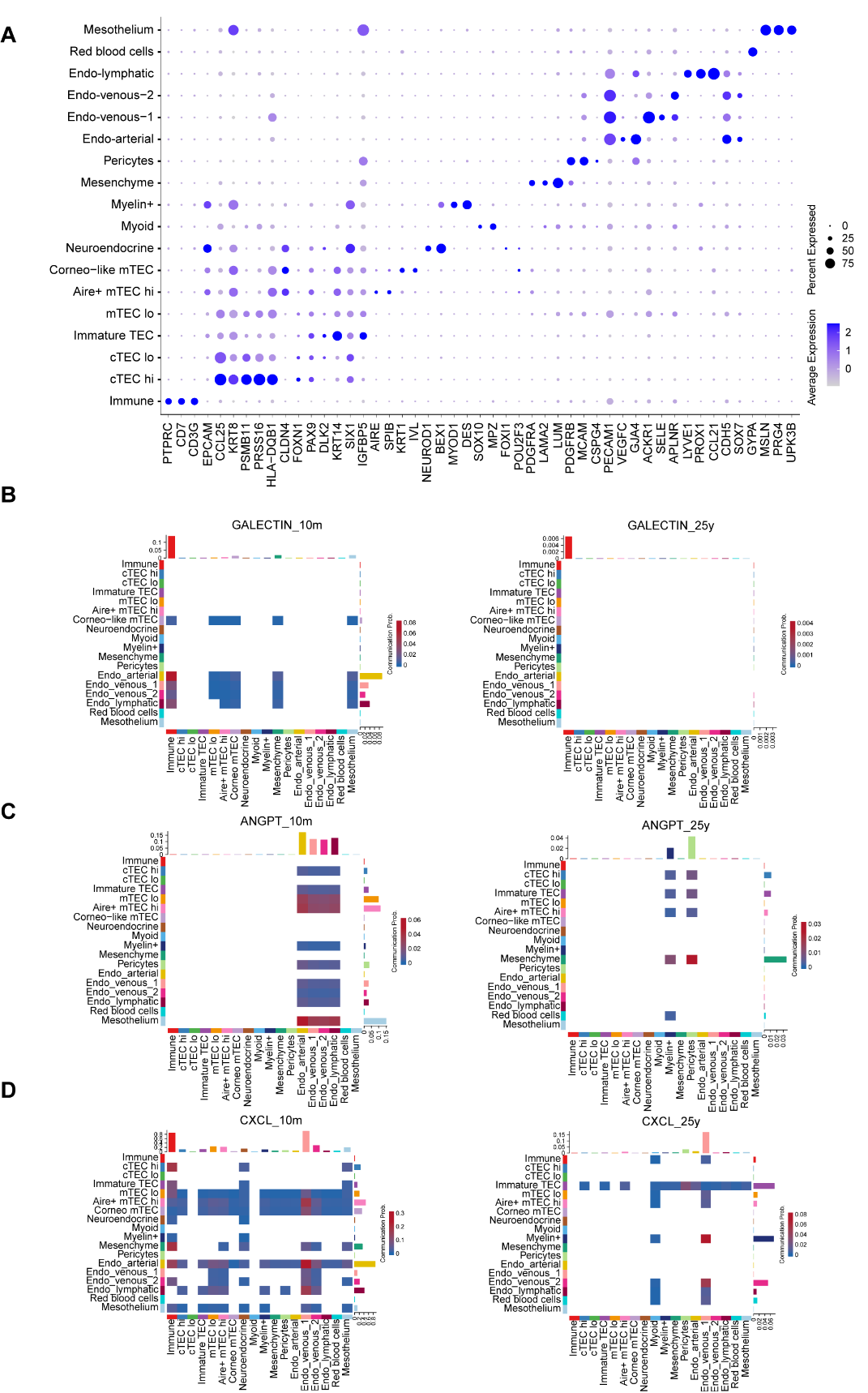


**Supplementary Figure 4.** **(A)** Dot plot showing marker gene expression for annotated cell types. Heatmaps show outgoing and incoming signaling of GALECTIN **(B)**, ANGPT **(C)**, CXCL **(D)** associated with each cell population at different ages. X-axis is incoming signaling and Y-axis is outgoing signaling.


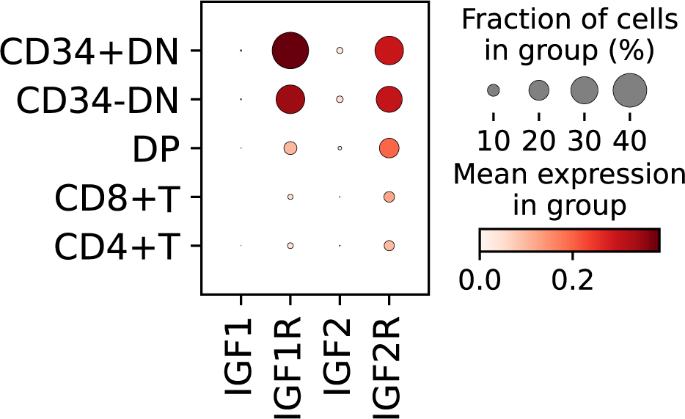


**Supplementary Figure 5.** Dot plot showing marker gene expression for annotated cell types.
